# Studies of the Impact of the *Bifidobacterium* Species on Inducible Nitric Oxide Synthase Expression and Nitric Oxide Production in Murine Macrophages of the BMDM Cell Line

**DOI:** 10.1007/s12602-023-10093-3

**Published:** 2023-05-25

**Authors:** Agnieszka Zabłocka, Dominika Jakubczyk, Katarzyna Leszczyńska, Katarzyna Pacyga-Prus, Józefa Macała, Sabina Górska

**Affiliations:** Laboratory of Microbiome Immunobiology, Hirszfeld Institute of Immunology and Experimental Therapy, Polish Academy of Sciences, Wroclaw, Poland

**Keywords:** *Bifidobacterium*, Probiotics, Inducible nitric oxide synthase, Nitric oxide, Kinase

## Abstract

*Bifidobacterium* species are one of the most important probiotic microorganisms which are present in both, infants and adults. Nowadays, growing data describing their healthy properties arise, indicating they could act at the cellular and molecular level. However, still little is known about the specific mechanisms promoting their beneficial effects. Nitric oxide (NO), produced by inducible nitric oxide synthase (iNOS), is involved in the protective mechanisms in the gastrointestinal tract, where it can be provided by epithelial cells, macrophages, or bacteria. The present study explored whether induction of iNOS-dependent NO synthesis in macrophages stems from the cellular action of *Bifidobacterium* species. The ability of ten *Bifidobacterium* strains belonging to 3 different species (*Bifidobacterium longum*, *Bifidobacterium adolescentis*, and *Bifidobacterium animalis*) to activate MAP kinases, NF-κB factor, and iNOS expression in a murine bone-marrow-derived macrophages cell line was determined by Western blotting. Changes in NO production were determined by the Griess reaction. It was performed that the *Bifidobacterium* strains were able to induce NF-қB-dependent iNOS expression and NO production; however, the efficacy depends on the strain. The highest stimulatory activity was observed for *Bifidobacterium animalis* subsp. *animals* CCDM 366, whereas the lowest was noted for strains *Bifidobacterium adolescentis* CCDM 371 and *Bifidobacterium longum* subsp. *longum* CCDM 372. Both TLR2 and TLR4 receptors are involved in *Bifidobacterium*-induced macrophage activation and NO production. We showed that the impact of *Bifidobacterium* on the regulation of iNOS expression is determined by MAPK kinase activity. Using pharmaceutical inhibitors of ERK 1/2 and JNK, we confirmed that *Bifidobacterium* strains can activate these kinases to control iNOS mRNA expression. Concluding, the induction of iNOS and NO production may be involved in the protective mechanism of action observed for *Bifidobacterium* in the intestine, and the efficacy is strain-dependent.

## Introduction

*Bifidobacterium* represents 40–80% bacterial genus of the total infants’ gut microbiota [1]; therefore, as the predominant genera in the gut of breastfeeding infants, they seem to be essential for human well-being. Their interactions with the host are tiered. Rabe et al. [2] reported that early colonization with bifidobacterial strains enhances T cell maturation in later childhood, increases a memory CD45RO + CD4 + T cell population, and participates in a stronger mitogen-induced cytokine response (IL-13, IL-5, IL-6, and TNF) by mononuclear cells. Wu et al. [3] indicated that they have an impact on the Th1/Th2 balance among healthy infants and enhance Th1 immune response through IFN-γ secretion in early life. It was shown that bifidobacterial gut colonization is delayed among premature babies which results in an increased likelihood of developing inflammatory diseases such as necrotizing enterocolitis (NEC) [4]. However, the bacterial footprint is much wider, and the impoverishment of the bifidobacterial population is linked with delay in neurodevelopment among preterm infants [5], obesity in childhood [6], eczema in childhood [7], pediatric inflammatory bowel disease (IBD) [8], and others. *Bifidobacterium* species are crucial also in further life. It was observed that the *Bifidobacterium* community undergoes changes depending on age. Kato et al. [9] reported that *B. breve* is predominant in children under 3 years old, *B. adolescentis* and *Bifidobacterium catenulatum* expansion is linked with diet modification after weaning, *Bifidobacterium bifidum* accompany the human during the whole life, whereas some of the *B. longum* group bacteria, *B. adolescentis* group, and *Bifidobacterium dentium* are characteristic for centenarians [9]. The abundance of *Bifidobacterium* strains changes with age [10]. The diversity reduction results from the weakening of bacterial adhesion to the intestinal mucosa. However, it is still unclear which stimuli (host-or microbiota-dependent) lead to this phenomenon [10]. Clinically, it is repeatedly emphasized that the depletion of *Bifidobacterium* strains is linked with health deterioration and an increased likelihood of developing chronic inflammatory diseases, such as IBD [11, 12]. It was shown that patients with active IBD characterize a significant decrease in the population of those bacteria [13, 14]. A rich *Bifidobacterium* community is associated with improving immune response, protecting pathogens’ adhesion, reinforcing of intestinal epithelial barrier function, and others [15, 16]. The supplementation with the proper bifidobacterial strains attenuates the inflammation and reduces symptoms of many diseases such as allergy, asthma, inflammatory bowel disease, celiac, and cancer [17–22]. However, despite advanced research, the knowledge about the exact path of host-bifidobacterial cell interaction is still blurry. Moreover, the data available focuses mainly on the epithelial barrier, dendritic cells, and lymphocytes’ response in various models [23]. The data concerns other kinds of cells and their relation to bifidobacterial strains is still in the minority, and this pertains, for instance, the macrophages. However, available sources indicate that *Bifidobacterium* and its derivatives affect the phenotype and function of macrophages [24], can modulate the pro-inflammatory response after LPS stimulation [25], and promote their proliferation and phagocytosis [26].

Macrophages compose a diversified population of mononuclear cells, which is present in all types of tissue. They belong to the innate immune system, and their primary role, pronounced by pathogen combat, tissue injury reparation, and maintenance of homeostasis, strongly depends on the external actuation [27]. According to the generally accepted assumption, in response to environmental stimuli, macrophages differentiate into M1 or M2 types. The phenotype M2 represents the anti-inflammatory population and arises by Th2-related cytokines stimulation, such as IL-4 and IL-13. This group participates in wound healing, tissue remodeling, and termination of inflammatory processes [28]. However, the M1 subtype has different characteristics. It is formed as a result of Th1-related factors (e.g., IFN-γ, TNF-α) and LPS. It possesses proinflammatory properties expressed, among others, by enhancing the anti-microbial and antitumor response and producing reactive oxygen and nitrogen species (ROS and RNS respectively) [28]. M1-macrophages are also an important source of nitric oxide (NO), a particle named the “molecule of the year in 1992.” Despite the passage of years, the interest in NO is not dwindling, mainly due to its multi-functionality. In this context, nitric oxide’s role in human health is especially interesting.

NO, synthesized from L-arginine by NOS (NO synthase), is an endogenous gas, which fulfills many biological functions, including metabolic regulator, signaling, and effector molecule [29, 30]. NOS has a few sources in the human body and occurs in 3 isoforms: brain or neuronal (nNOS), inducible (iNOS), and endothelial (eNOS). eNOS and nNOS are released constitutively, whereas iNOS is induced under pro-inflammatory conditions and oxidative stress, by, among others, macrophages [31]. In physiological conditions, nitric oxide produced by endothelial cells is a vasodilating factor; it regulates the local cells grown and protects from the platelets’ adhesion to the surface of the endothelium [30]. The high level of endothelial NO disturbs homeostasis, changes vascular permeability, and induces muscle relaxation. Its low level is a risk factor for cardiovascular diseases and atherosclerosis [32]. The significance of NO is highlighted in the human gastrointestinal tract, which is the major source of NO [33]. Nitric oxide can be provided by neurons (enteric nervous system), endothelial cells, macrophages, and even bacteria. The role of NO is diversified. In physiological conditions; it enables secretion, digestion, and motility of the gut. It maintains mucus production and water transport. However, with the destroyed gut tissue, the inflammatory response takes place, which could cause disturbed NO production. A high level of NO induces apoptosis, mutagenesis, DNA damage, and the function of the mitochondria in the cell [34].

The present study aimed to determine the potential of ten *Bifidobacterium* strains to induce NO in murine bone-marrow-derived macrophages cell line and decipher the signaling pathway involved in the induction of iNOS-dependent NO synthesis.

## Materials and Methods

### Materials

High-glucose Dulbecco’s modified Eagle’s medium (DMEM), trypsin + EDTA mixture, and phosphate-buffered saline (PBS) (pH 7.4) were prepared in the Laboratory of General Chemistry of the Institute of Immunology and Experimental Therapy, PAS (Wroclaw, Poland). Bacterial lipopolysaccharide (LPS) from *Escherichia coli* (serotype 055:B5), 3-(4,5-dimethylthiazol-2-yl)-2–5-diphenyltetrazolium bromide (MTT), and Tween-20 were purchased from Sigma (New York, NY, USA). L-glutamine and antibiotics (penicillin/streptomycin mixture) were purchased from BioWest (Nuaillé, France). Fetal bovine serum (FBS) was purchased from EURx Ltd (Gdańsk, Poland). Reagents for SDS-PAGE and protein marker were purchased from Bio-Rad (Hercules, CA, USA). N-(1-naphthyl)-ethylenediamine was purchased from Serva Feinbiochemica (Heidelberg, Germany). Sulfanilamide, sodium nitrite, orthophosphoric acid, KH_2_PO_4_, and K_2_HPO_4_ were purchased from Avantor (Gliwice, Poland). Alkaline phosphatase-conjugated anti-rabbit IgG antibodies were from Cell Signaling Technology (Danvers, MA, USA). Anti ERK1/2, anti-phospho ERK1/2, anti-JNK, anti-phospho JNK, anti-NF-қB, and anti-phospho NF-қB p65 (Ser 536) monoclonal antibody, anti-STAT 3, and U0126 inhibitor were obtained from Cell Signaling Technology (Danvers, MA, USA). anti-iNOS monoclonal antibody was from Santa Cruz Biotechnology (Dallas, TX, USA). SP600125 inhibitor was obtained from Med Chem Express (New York, NY, USA). 5-bromo-4-chloro-3-indolyl phosphate disodium salt (BCIP) and nitro-blue tetrazolium (NBT) were from Carl Roth GmbH (Karlsruhe, Germany).

### Bacterial Strains

Ten different *Bifidobacterium* strains (Table 1) were obtained from the Collection of Dairy Microorganisms (Laktoflora, Milcom, Tábor, Czech Republic) and grown as described by Pyclik et al. [35]. Before tests, strains were centrifuged (4300 × g, 8 min, RT), washed twice, and next resuspended in phosphate-buffered saline (PBS, pH 7.4). Bacterial cell numbers were determined by measuring the value at OD_600_ in spectrophotometric microplate counter (BioTek, Winooski, Vermont, USA), which was related to the number of CFUs on MRS agar plates with 0.05% L-cysteine after 48 h of growth under anaerobic conditions (0.328% O_2_ and 9.84% CO_2_). Additionally, all strains were stored in an MRS broth medium with 0.05% L-cysteine and 20% glycerol at − 80 °C.

**Table 1:** *Bifidobacterium* species

***Bifidobacterium *species**	**Abbreviation**
* Bifidobacterium adolescentis* CCDM 373	373
* Bifidobacterium longum* subsp. *longum* CCDM 372	372
* Bifidobacterium adolescentis* CCDM 371	371
* Bifidobacterium adolescentis* CCDM 370	370
* Bifidobacterium longum* subsp*. infantis* CCDM 369	369
* Bifidobacterium adolescentis* CCDM 368	368
* Bifidobacterium longum* subsp. *longum* CCM 7952	367
* Bifidobacterium animalis* subsp. *animalis* CCDM 366	366
* Bifidobacterium longum* subsp. *longum* CCDM 219	219
* Bifidobacterium animalis* subsp. *animalis* CCDM 218	218

### Cell Culture

A macrophage cell line derived from the bone marrow of wild type of mice (BMDM)(NR-945), a macrophage cell line derived from the bone marrow of TLR2 knockout mice (BMDMTLR2-)(NR-9457), and a macrophage cell line derived from the bone marrow of TLR4 knockout mice (BMDMTLR4-)(NR-9458) were obtained from BEI Resources, NIAID, NIH. The cell lines were maintained in Dulbecco’s modified Eagle’s medium (DMEM) supplemented with 10% FBS, antibiotics, and 3% L-glutamine according to the manufacturer’s instructions. Cells were grown under standard conditions in a humidified incubator at 37 °C in an atmosphere of 95% air and 5% CO_2_. Adherent cells from confluent cultures were detached, centrifuged at 150 × g for 10 min, and suspended in a complete culture medium.

### MTT Test

The viability of BMDM cells was determined using MTT colorimetric assay [36]. Briefly, BMDM cells were seeded onto a 96-well plate (1 × 10^4^/well) and incubated overnight in 5% CO_2_/95% air at 37 °C, in a 10% FBS complete medium. The next day, the medium was replaced with a fresh one, and the cells were stimulated with a particular *Bifidobacterium* strain (0.5 × 10^8^ CFU/mL). Untreated BMDM cells were used as a negative control. After 24 h, the supernatants were removed, and the cells were incubated with an MTT reagent (5 mg/mL in PBS pH 7.4) for 4 h at 37 °C. Next, 100 µL of DMSO was added to the plate to dissolve the formed formazan crystals. The absorbance was measured using an EnSpire™ 2300 microplate reader (PerkinElmer, MA, USA) at 570 nm. Viability was expressed as a percentage of living cells versus control untreated cells (100%).

### Assay to Nitrite/Nitrate Generation

- BMDM cells were seeded on a 12-well plate at 1 × 10^6^ cells/mL density and cultured in a 10% FBS complete medium for 24 h in 5% CO_2_/95% air. The next day, the medium was replaced and particular *Bifidobacterium* strains (0.5 × 10^8^ CFU/mL/well) were added to the cells, separately. LPS from *E. coli* (serotype O55B5, 1 µg/mL) was used as a positive control, while untreated BMDM cells were used as a reference control. Since NO is synthesized by inducible NOS, selective iNOS inhibitor S-MIU (10 µM) was used to check the specificity of NO production. Additionally, to determine the impact of ERK 1/2 and JNK kinases on the regulation of NO production, BMDM cells were pre-incubated for 1 h with selective kinase inhibitors: U0126 (20 µM)(for ERK1/2) and SP600125 (10 µM)(for JNK) and then stimulated with particular *Bifidobacterium* strains (0.5 × 10^8^ CFU/mL). After 24 h of incubation in a humidified atmosphere enriched with 5% CO_2_ at 37 °C, cell culture supernatants were centrifuged at 6000 × g for 5 min at RT, and the resultant cell-free supernatant was assayed for the determination of nitric oxide concentration.
- To determine whether selected *Bifidobacterium* strains activate BMDM cells by TLR2 and/or TLR4 receptor, TLR2-deficient and TLR4-deficient BMDM cell lines (BMDM TLR2- and BMDM TLR4-, respectively) were used. BMDM cells were plated onto a 12-well plate at 1 × 10 cells/mL density and cultured in10% FBS complete medium. Particular *Bifidobacterium* strains (0.5 × 10^8^ CFU/mL/per strain) were added to the cells separately, as potential inducers of nitric oxide. Untreated BMDM cells were used as a negative control. LPS (1 µg/ml), activating TLR4 but not TLR2, was used as a reference sample. After 24 h of incubation in a humidified atmosphere enriched with 5% CO_2_ at 37 °C, cell culture supernatants were centrifuged at 6000 × g for 5 min at RT, and the resultant cell-free supernatant was assayed for determination of nitric oxide concentration.
- To determine the ability of *Bifidobacterium* to NO self-production, individual *Bifidobacterium* strains (0.5 × 10^8^ CFU/mL/per strain) were incubated for 24 h in a 10% FBS complete medium in a humidified atmosphere enriched with 5% CO_2_ at 37 °C. Next, the supernatants were centrifuged at 6000 × g for 5 min at RT, and the resultant cell-free supernatant was assayed for nitric oxide level.

### NO Determination

NO production was measured by testing the nitrite concentration in the supernatants of cultured BMDM cells after 24-h incubation with *Bifidobacterium* using the colorimetric method with Griess reagent [37]. Briefly, 100-μL samples of cell supernatants were incubated with an adequate amount of Griess reagent (0.1% N-(1-naphthyl)-ethylenediamine and 1% sulfanilamide in 5% phosphoric acid). After 10-min incubation at room temperature (RT), the absorbance at 570 nm was measured. The concentration of nitrite was determined by comparison with the NaNO_2_ standard curve (0 to 75 μM).

### Western Blotting

BMDM cells (1 × 10^6^ cells/mL) were seeded onto 12-well culture plates and incubated with *Bifidobacterium* (0.5 × 10^8^ CFU/mL) for 24 h in 5% CO_2_/95% air for activation of ERK 1/2, JNK, NF-қB, STAT3, and iNOS expression. After stimulation, the bacteria were rinsed with PBS, next, the cells were lysed in RIPA buffer (150 mM NaCl, 50 mM Tris–HCl pH 7.5, 5 mM EDTA, 1% Triton X–100, 0.1% SDS, 0.5% deoxycholate) supplemented with protease inhibitor cocktail (Roche, Basel, Switzerland), 1 mM NaF and 2 mM Na_3_VO_4_, keeping on ice for 30 min. Lysates were centrifuged at 14,000 × g for 10 min at 4 °C, and then protein content was determined by the bicinchoninic acid (BCA) method using BSA as a standard. The 30 µg of protein samples was separated on 4–12% sodium dodecyl sulfate (SDS)–polyacrylamide gel (TXG Fast Cast Acrylamide solutions (Bio-Rad, Hercules, CA, USA) and next transferred to a nitrocellulose membrane. The membrane was blocked (Tris–HCl buffer, pH 7.0, 5% Tween 20 (TBST) and 5% nonfat dried milk) for 1 h at RT and then probed overnight at 4 °C with primary antibodies diluted in TBST with 5% BSA: anti-iNOS (1:1000), anti-β-actin (1:1000), anti-ERK 1/2 (1:1000), and anti-phospho ERK 1/2 (1:1000), anti- JNK (1:1000), anti-phospho JNK (1:1000), anti-NF-kB (1:1000), anti-phospho NF-kB p65 (1:1000), or anti- STAT3 (1:1000). Next, the membrane was incubated for 1 h at RT using secondary antibodies conjugated with alkaline phosphatase (1:10,000 in TBST with 5% BSA) according to standard procedure. Immunocomplexes were visualized using an NBT/BCIP substrate and photographic documentation was done using the Molecular Imager ChemiDoc MP Imaging System (Bio-Rad, Hercules, CA, USA).

### Statistical Analysis

Statistical analysis was performed using GraphPad Prism 9.5.1 Software (San Diego, CA, USA). Comparisons between groups were based on one sample *t*-test or one-way ANOVA test. The value of *p ≤ 0.05, **p ≤ 0.001, ***p ≤ 0.0001, and ****p < 0.0001 was considered statistically significant.

## Results

### *Bifidobacterium* Upregulates BMDM Viability, iNOS Expression, and NO Production Differentially Depending on the Strain

**Fig. 1:**
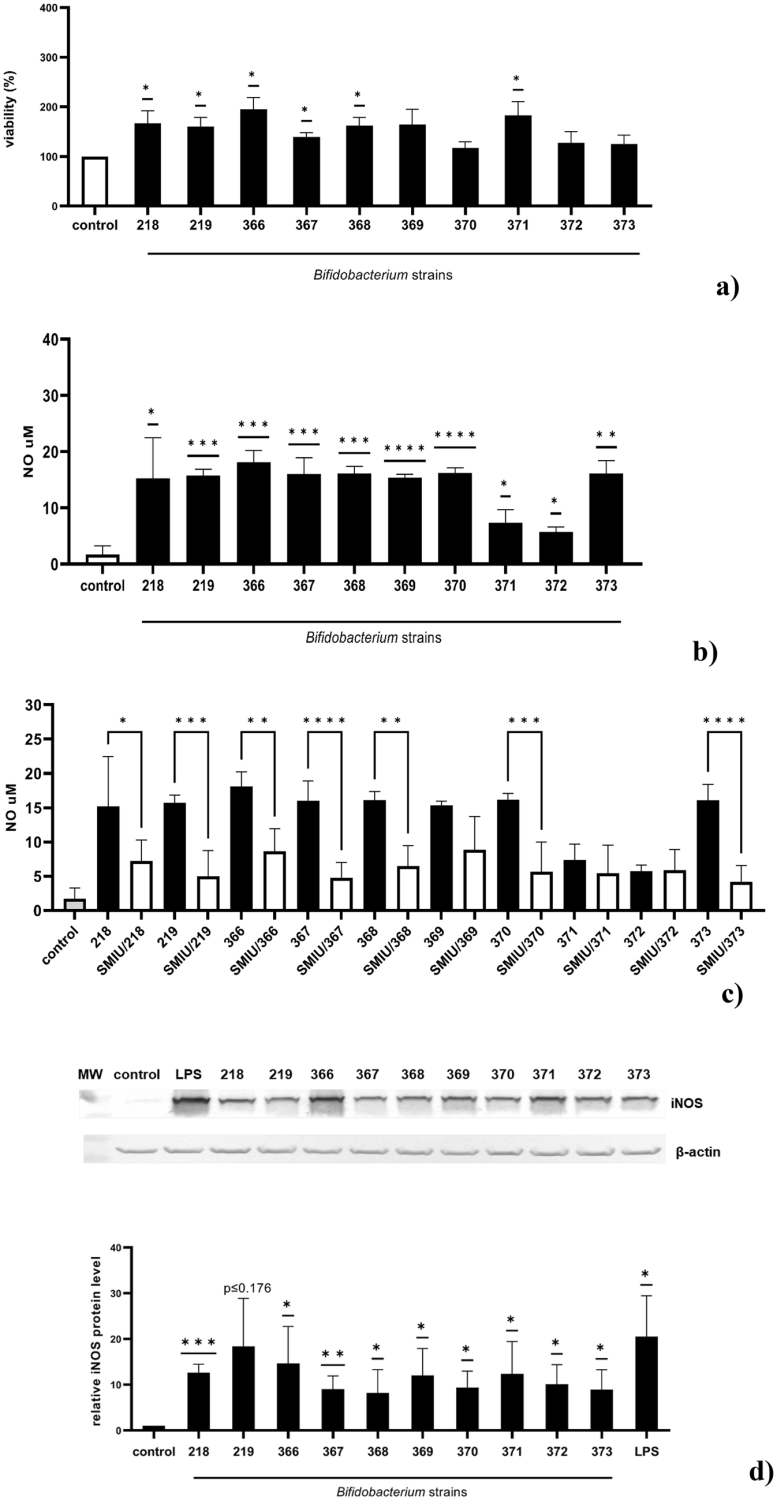
The effect of *Bifidobacterium *strains on the viability of BMDM cells (**a**), nitric oxide production (**b**, **c**), and iNOS expression (**d**). **a** BMDM cells (1 × 10^5^/mL) were seeded in 96-well plates in DMEM + 10% FBS and incubated overnight at 37 °C in 5% CO_2_/95% air. Next, the cells were exposed to *Bifidobacterium* strains (0.5 × 10^8^ CFU/mL) for 24 h. Cell viability was assessed with an MTT assay. Non-stimulated cells (control) were used as a reference sample. The results represent at least three independent experiments and data are presented as mean ± SD. One sample *t*-test was used to examine the mean differences between samples. *p ≤ 0.05, **p ≤ 0.001 vs control. **b** BMDM cells (1 × 10^6^/mL) were cultured with a particular *Bifidobacterium* strain (0.5 × 10^8^ CFU/mL) for 24 h in 5% CO_2_/95% air. Thereafter, supernatants were collected, and a level of NO was detected by the Griess reaction. The results represent at least three independent experiments and data are presented as mean ± SD. One sample *t*-test was used to examine the differences between examined samples; *p ≤ 0.05, **p ≤ 0.001, ***p ≤ 0.0001, and ****p < 0.0001 vs control. **c** To check the specificity of NO production, BMDM cells were firstly pretreated with selective iNOS inhibitor S-MIU (10 µM) for 1 h, and next cultured with a particular *Bifidobacterium* strain (0.5 × 10^8^ CFU/mL) for 24 h. Thereafter, supernatants were collected, and a level of NO was detected by the Griess reaction. The results represent at least three independent experiments and data are presented as mean ± SD. One-way ANOVA test was used to examine the differences between *Bifidobacterium-*treated BMDM cells in the presence (white columns) and absence (black columns) of S-MIU; *p ≤ 0.05, **p ≤ 0.001, ***p ≤ 0.0001, and ****p < 0.0001 vs particular *Bifidobacterium* strain alone. **d** BMDM cells were treated with a particular *Bifidobacterium* strain *(*0.5 × 10^8^ CFU/mL) or left untreated for 24 h (control). LPS (1 µg/mL) was used as a positive control. The level of iNOS protein was detected in cell lysates by immunoblotting using monoclonal anti-iNOS antibodies. Fold change in iNOS levels was compared to *β*-actin. Results represent at least three independent experiments, and data are presented as mean ± SD. One sample *t*-test was used to examine the mean differences between samples. *p ≤ 0*.*05, ** *p* ≤ 0.01, ***p ≤ 0.001 vs control

We used the MTT assay to monitor the viability of BMDM cells. It was observed that the *Bifidobacterium* strains studied were not toxic to the BMDM cells. Additionally, after 24 h of incubation, a significant increase in the number of BMDM viable cells was observed in the presence of *Bifidobacterium* strains 218, 219, 366, 367, 368, and 371 tested, compared to the control untreated cells. The highest increase in cell viability, reaching up to 166,8%, 195%, and 183% in response to 218, 366, and 371 strains respectively, was observed (Fig. 1a).

Nitric oxide is involved in the protective mechanisms of the gastrointestinal tract and may contribute to some of the beneficial, pro-healthy effects of probiotics [38, 39]. Hence, in the present study, the impact of *Bifidobacterium* on iNOS expression and NO production was determined. It was observed that viable *Bifidobacterium b*acteria in co-culture with murine BMDM macrophages induced the production and release of NO into the culture medium in a significant amount (Fig. 1b). This effect was strongly associated with the upregulation of iNOS expression in BMDM cells (Fig. 1d). All *Bifidobacterium* strains studied upregulated the NO production, and the amount of released NO remained in a stable range of 5–18 µM (Fig. 1b). Simultaneously, the increase in iNOS expression level vs untreated control cells was indicated (12.6-fold for 218, 13.4-fold for 219, 9-fold for 367, 8.1-fold for 368, 12-fold for 369, 9.3-fold for 370, and 8.9-fold for 373). The highest stimulatory activity was observed for strain 366 (18.11 µM of NO vs 18.1-fold of iNOS expression). The level of NO production was abolished in samples treated with *Bifidobacterium* in the presence of the selective iNOS inhibitor—S-MIU, indicating NO release is strictly linked to the induction of iNOS expression (Fig. 1c).

**Fig. 2:**
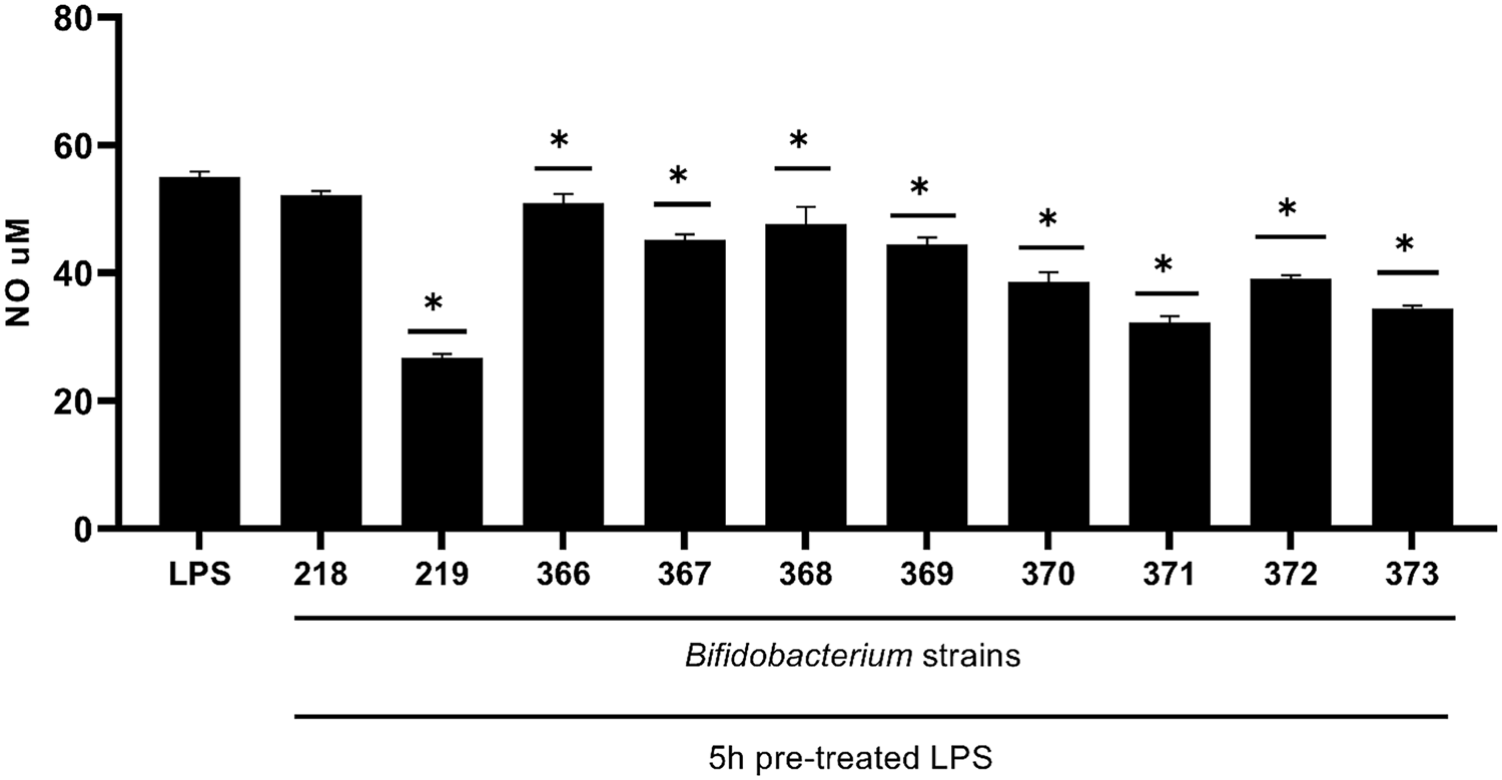
Effect of *Bifidobacterium* on nitric oxide production in LPS-pretreated BMDM cells

The anti-inflammatory potential of *Bifidobacterium* strains was also evaluated in the LPS-stimulated macrophages. BMDM cells were pre-incubated with LPS (1 µg/ml) for 5 h to induce expression of proinflammatory inducible NOS responsible for nitric oxide production. Interestingly, all tested *Bifidobacterium* strains, excluding 218, were able to diminish LPS-induced NO production in BMDM cells; nevertheless, the efficacy was strain-dependent. The highest inhibitory activity was observed in *Bifidobacterium* strains 219, 371, and 373, which were able to reduce NO production up to 26.7 µM, 32.2 µM, and 34.3 µM respectively, compared to LPS alone (54.9 µM) (Fig. 2).

BMDM cells (1 × 10^6^/mL) were pretreated with LPS (1 µg/mL) for 5 h, and the next particular *Bifidobacterium *strain (0.5 × 10^8^ CFU/mL) was added. LPS alone (1 µg/mL) was used as a reference sample. After 24 h incubation, the supernatants were collected and the level of NO was detected by the Griess reaction. The results represent three independent experiments, and data are presented as mean ± SD. A one-way ANOVA test was used to examine the differences between samples. *p ≤ 0.05 vs LPS alone.

### *Bifidobacterium* Strains Upregulated iNOS Expression and NO Production by Targeting the MAPK/NF-қB Signaling Pathway, and TLR2 and TLR4 Activation

To decipher, which cellular mechanisms are responsible for controlling NO production in macrophages of the BMDM cell line treated with *Bifidobacterium*, their impact on Toll-like receptors 2 and 4, the MAP kinases-dependent signaling pathway, and the transcription factor NF-қB and STAT3 activation were examined.

### TLR2 and TLR4 Receptors are Involved in *Bifidobacterium*-Induced Macrophage Activation and NO Production

**Fig. 3:**
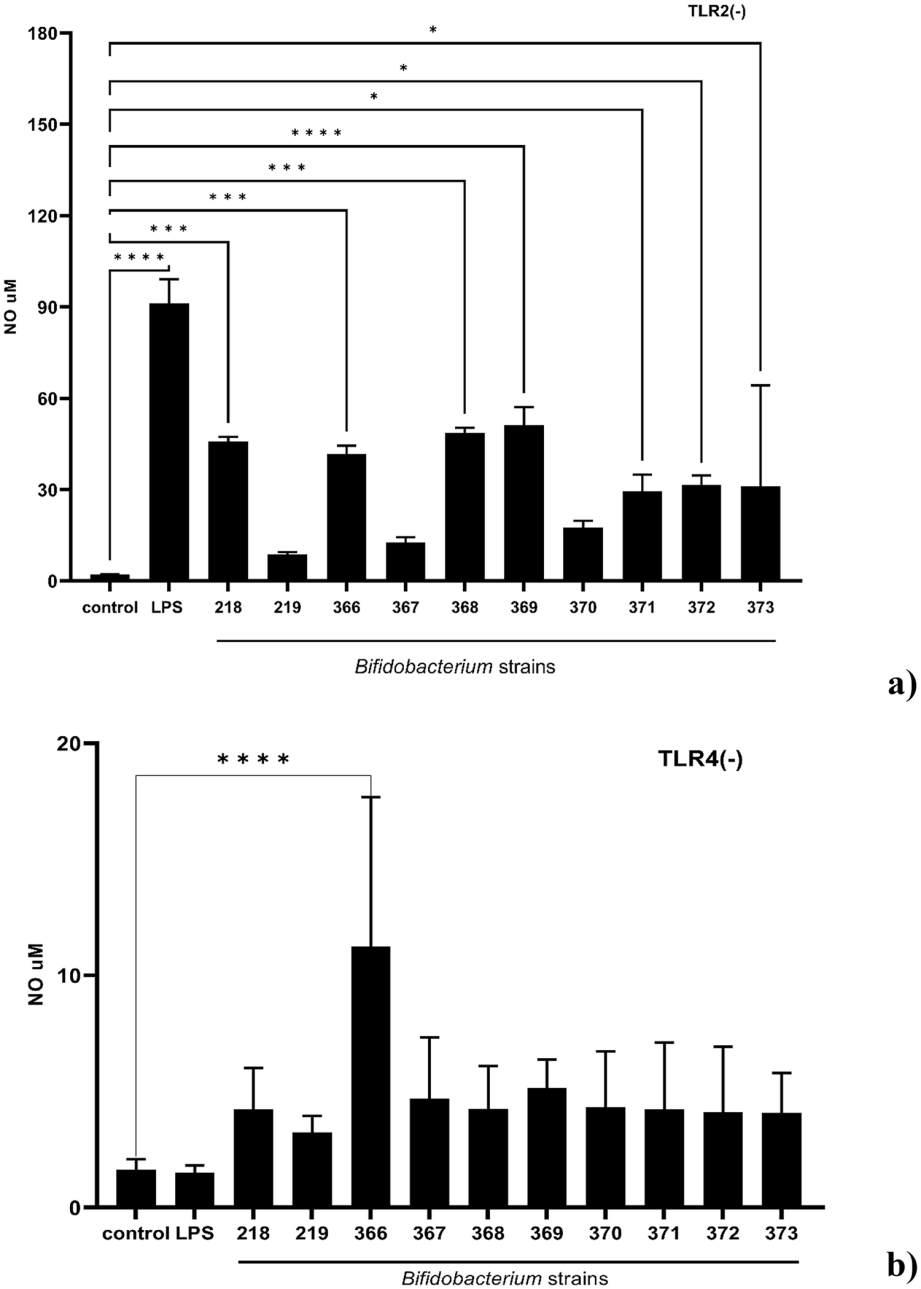
Effect of *Bifidobacterium* on TLR2 and TLR4 receptors activation and nitric oxide production in BMDM cells

The macrophage cell line derived from the bone marrow of TLR2 knockout mice (BMDM TLR2-) (Fig. 3a) and the macrophage cell line derived from the bone marrow of TLR4 knockout mice (BMDM TLR4-) (Fig. 3b) was used to determine the role of TLR4, and TLR2 receptors in *Bifidobacterium-*induced NO production in macrophages. The cells were incubated with *Bifidobacterium* for 24 h. LPS (1 μg/mL), activating TLR4 but not TLR2 receptor, was used as a relative control sample. As expected, the LPS alone significantly activated NO production in TLR2-deficient BMDM cells, but not in TLR4-deficient BMDM cells. However, both TLR2 and TLR4 receptors can be activated by *Bifidobacterium* strains 219, 367, and 370 to induce NO production. Interestingly, strain 366 triggered NO production in both TLR2 and TLR4–deficient BMDM cells. The remaining strains tested: 218, 368, 369, 371, 372, and 373 needed the presence of TLR4 receptor to activate signaling pathways resulting in the production of nitric oxide.

Macrophage cell line derived from the bone marrow of TLR2 knockout mice (BMDM TLR2-) (a) and macrophage cell line derived from the bone marrow of TLR4 knockout mice (BMDM TLR4-) (b) (1 × 10^6^/mL) were incubated with particular *Bifidobacterium* strains (0.5 × 10^8^ CFU/mL), or left untreated (control). LPS (1 µg/ml) was used as a reference sample. After 24 h incubation, the supernatants were collected and the level of NO was detected by the Griess reaction. Results represent at least 3 independent experiments and data are presented as mean ± SD. A one-way ANOVA test was used to examine the differences between samples. ∗ *p* ≤ 0:05, ***p ≤ 0.001, ****p ≤ 0.0001 vs control.

### *Bifidobacterium* Activates both MAPK Kinases and Transcription Factor NF-қB

**Fig. 4:**
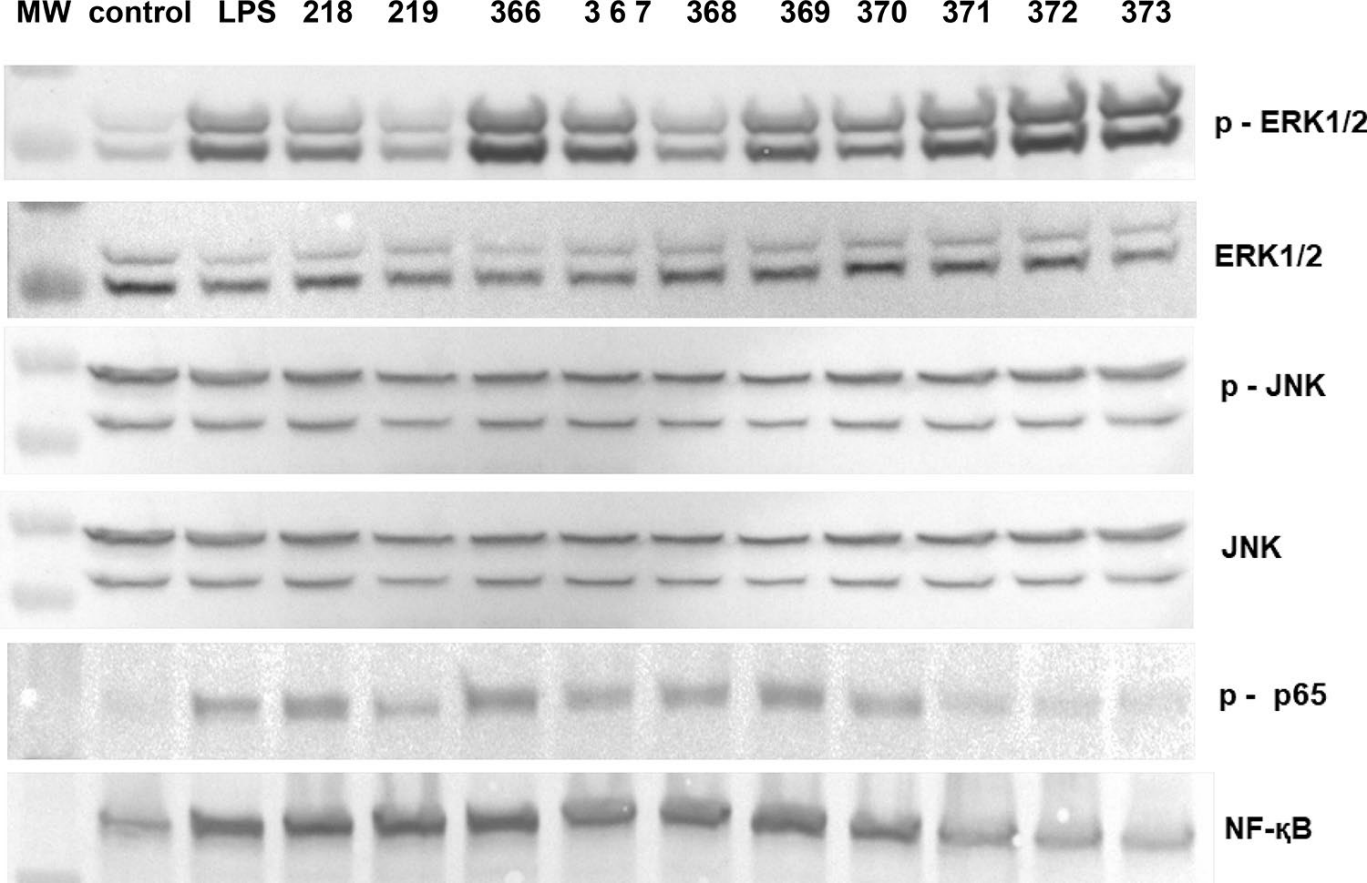
Activation of ERK 1/2 kinases, JNK kinase, and NF-қB factor in BMDM macrophages stimulated with *Bifidobacterium*

Activation of TLRs links to the increased upregulation of MAPK kinases, transcription factors NF-қB and STAT3, resulting in iNOS expression [40, 41]. Therefore, the impact of *Bifidobacterium* on MAPK kinases (ERK 1/2 and JNK) and NF-қB and STAT3 transcription factor activation in BMDM cells was studied. Immunoblot analysis revealed that all tested *Bifidobacterium* strains induce a significant increase in the phosphorylation of ERK 1/2, and JNK kinases compared to control, nontreated cells (Fig. 4). Next, the activation of NF-қB in macrophages was evaluated by NF-қB p65 phosphorylation detection. As shown in Fig. 4, all strains of *Bifidobacterium* studied significantly upregulated NF-қB p65 phosphorylation at a comparable level range of 2.4 to 4.0-fold compared to control nontreated cells. The impact of *Bifidobacterium* on STAT3 activation was also determined; however, no activating effect was observed (data not shown).

Mouse BMDM macrophages were cultured in the presence of particular *Bifidobacterium* strains (0.5 × 10^8^ CFU/mL) or left nontreated (control) for 24 h at 37 °C. LPS (1 µg/ml) was used as a reference sample. Whole-cell lysates were prepared and analyzed by immunoblotting using specific Abs to the basic and phosphorylated ERK 1/2, JNK, and NF-қB p65 (*n* = 3–5). Immunocomplexes were visualized in Molecular Imager ChemiDoc MP Imaging System (Bio-Rad, Hercules, CA, USA). Representative blots were shown.

**Fig. 5:**
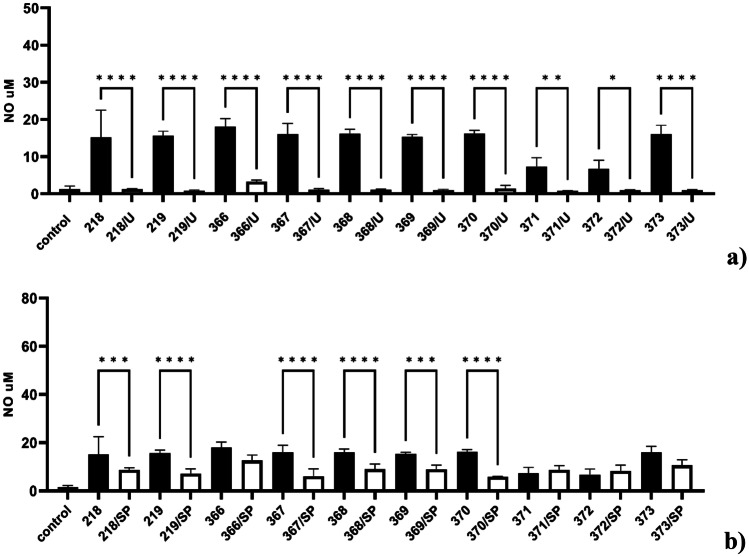
Impact of ERK 1/2 inhibitor U0126 (**a**) and JNK inhibitor SP600125 (**b**) on NO production in BMDM macrophages. Mouse BMDM macrophages were firstly preincubated for 1 h with ERK 1/2 inhibitor U0126 (U)(20 µM) or JNK inhibitor SP600125 (SP)(10 µM) and then cultured with particular *Bifidobacterium* strain (0.5 × 10^8^ CFU/mL) for 24 h at 37 °C. Non-stimulated cells (control) were used as a negative control. Thereafter, supernatants were collected, and a level of NO was detected by the Griess reaction. The results represent at least three independent experiments and data are presented as mean ± SD. A one-way ANOVA test was used to examine the differences between *Bifidobacterium-*treated BMDM cells in the presence and absence of kinase inhibitors. *p ≤ 0.05, **p ≤ 0.001, ***p ≤ 0.0001, and ****p < 0.0001 vs *Bifidobacterium* strain alone

To assess whether the iNOS-dependent NO production is under the control of ERK 1/2 and/or JNK kinases, the BMDM cells were firstly pre-treated with a pharmacological inhibitor of ERK 1/2-U0126 or JNK-SP600125 and then exposed to *Bifidobacterium* at 37 °C for 24 h. The level of NO released to the supernatants was determined by the Griess method. Pretreatment of BMDM cells with ERK 1/2 inhibitor, U0126, was associated with a decrease in NO level comparable to control, nontreated cells (Fig. 5a). The pre-application of SP600125 (JNK-specific inhibitor) significantly inhibited NO production in response to the *Bifidobacterium* strains 218, 219, 367, 368, 369, and 370. There was no significant difference in the level of NO induced by strains 366, 371, 372, and 373 (Fig. 5b). These findings strongly suggest that activation of ERK 1/2 and JNK-dependent signaling pathway in macrophages is mainly related to the particular *Bifidobacterium* strain-induced iNOS expression and NO production.

## Discussion

Gut homeostasis is arranged by the cooperation between the immune system, the enteric nervous system, and intestinal microbiota including bacteria, viruses, and fungi [42]. Within the immune system, dendritic cells, lymphoid cells, and macrophages participate in the regulation of gut function [43].

*Bifidobacterium* strains are widely used as probiotics, which are defined as “live microorganisms which, administered in adequate amounts confer a health benefit on the host” [44, 45]. They have become one of the main research interests due to their potential immunoregulatory [46, 47] and anti-tumor activity on the host [22, 48]. It was shown that *Bifidobacterium* can activate NK cells, T cells, and also macrophages to produce a wide spectrum of mediators playing a pivotal role in the control of inflammation [49–51]. It is assumed that there are two possible paths of immunomodulatory action of *Bifidobacterium*. The first one could be connected with the direct interaction of *Bifidobacterium* with macrophages resident in the gut [52]. The second option could be the activation of parenteral macrophages (present in other tissues) via bacterial-released metabolites which in turn are absorbed into the bloodstream [46]. Nevertheless, the molecular mechanisms whereby *Bifidobacterium* modulates immune response mechanisms are still unclear and need to be intensively studied.

In the present study, the immunoregulatory activity of ten selected probiotic *Bifidobacterium* strains, demonstrated by the interactive potential with macrophage cells, was determined. The signaling pathways activated in *Bifidobacterium-*stimulated macrophages, providing nitric oxide production, were studied in detail. The experiments were performed on a macrophage cell line derived from the bone marrow of wild-type mice (BMDM), because of the important, and still unexplained role of macrophages in gut function regulation and protection. It is known, that macrophages, as the tissue-specific phagocytes, play an important role in the innate and adaptive immune response and constitute the first line of defense system against pathogenic microorganisms, such as bacteria or viruses and tumor cells [43, 53]. The intestine *lamina propria* contains a large number of macrophages playing a key role in killing invading microbes, eliminating dead cells, and contributing to mucosal healing, epithelial repair, and metabolism [54, 55].

It was shown in the present study that ten studied potentially probiotic *Bifidobacterium* strains (Table 1) are not toxic to BMDM macrophages and they may be considered safe. Additionally, strains 218, 219, 366, 267, 368, and 371 significantly increased their viability (Fig. 1a). It was also shown that *Bifidobacterium* strains tested display iNOS-dependent NO production, but the effect observed was diverse (Fig. 1b–d). Using a selective iNOS inhibitor S-MIU, significant inhibition of nitric oxide production took place, confirming that the observed effect is under iNOS control (Fig. 1c). Moreover, the level of NO accumulated in the strains’ culture broth was undetectable, which suggests that NO measured in the supernatants is directly produced in macrophages by iNOS. Nitric oxide, one of the most important inflammatory factors, possesses a pleiotropic activity including redox regulation, immunomodulation, and also the ability to kill or growth inhibition of tumor cells, bacteria, and parasites reaching the intestinal epithelium, and also their limiting gut colonization. NO can be also an important step in impeding viral replication in the infected hosts [56, 57]. The observed ability of the tested *Bifidobacterium* strains to induce the synthesis and production of nitric oxide in macrophages may therefore indicate their immunoregulatory potential in the gut. The ability of probiotics bacteria to stimulate macrophages to produce NO was also observed by Park et al. [58] and Han et al. [59] who demonstrated that exposure of RAW264.7 macrophages to *Bifidobacterium* resulted in a significant increase in NO production. Korhonen et al. [60] demonstrated that also probiotic *Lacticaseibacillus rhamnosus* induced iNOS-dependent NO synthesis in J774 mouse macrophages.

Next to regulatory activity, it was also observed in this study the potential anti-inflammatory properties of *Bifidobacterium *strains tested. When BMDM cells were firstly pre-treated with proinflammatory factor LPS for 5 h (to activate inflammatory response including, among others, the expression of iNOS) and next incubated with *Bifidobacterium*, the significant reduction in NO production was observed in all of *Bifidobacterium* strains studied, excluding 218. The most effective inhibitors of NO production were 219, 371, and 373, which downregulated the level of NO up to 52%, 41.3%, and 37.4%, respectively, compared to LPS alone. However, the mechanism of the anti-inflammatory activity of *Bifidobacterium* is unexplained yet. Because the probiotics can reduce inflammatory responses in both macrophage and intestinal epithelial cells [61, 62], we can hypothesize that result observed could be a consequence of the ability of *Bifidobacterium *to inhibit inflammatory-induced MAPK-NF-κB/iNOS-signaling pathway resulting in the downregulation of NO synthesis [62].

Transcriptional regulation of the *iNOS* gene is controlled by the NF-κB transcription factor, in both murine and human cells [63, 64]. It was discovered previously that some elements of the gut microbiota and/or their effector proteins can activate or suppress the transcription factor, nuclear factor kappa B (NF-κB)-dependent signaling pathway. NF-ĸB plays a critical role in determining the state of homeostasis or inflammation-associated dysbiosis in the gut [65]. It supports host-microbiota symbiosis and intestinal barrier integrity [66]. In the present study, we analyzed and compared the ability of *Bifidobacterium* to induce NF-κB activation in the context of iNOS expression regulation in murine BMDM cells. It was shown that all tested *Bifidobacterium* strains upregulated NF-κB phosphorylation, which is important for cytokines and iNOS expression control (Fig. 4). Our data are in line with Korhonen et al. [60], Miettinen et al. [67], and Park [58]. Likewise, Trapecar et al. [68] indicated that also *Lacticaseibacillus* subsp. affects intestinal epithelial cells and macrophages, leading to the upregulation of NF-κB p65 nuclear translocation. This nuclear translocation was connected with the ability of the commensal microbiome to activate the innate immune system against pathogens [69, 70].

Mitogen-activated kinases (MAPK) participate in NF-κB-dependent signal transduction in macrophages and regulate iNOS expression and NO synthesis [40, 41, 71]. There are two major MAPK subgroups: the extracellular signal-regulated kinases (ERK1/2) and the JNK/stress-activated protein kinases (JNK). In the present study, the *Bifidobacterium*-dependent upregulation of ERK 1/2 and JNK kinase phosphorylation (Fig. 4) but not kinase p38 (data not presented) were observed. The NO production in response to *Bifidobacterium* was significantly reduced after pretreatment of BMDM cells with ERK 1/2 inhibitor: U0126 or JNK inhibitor: SP600125 (Fig. 5), indicating the participation of both kinases in the iNOS expression and NO production. A comparable mechanism was also observed by Korhonen et al. [60]. In the model of J774 macrophages stimulated by *Lacticaseibacillus rhamnosus*, the iNOS-dependent NO production was under ERK 1/2 but not p38 kinase control.

Activation of MAP kinase pathways is critical in both pro-inflammatory and anti-inflammatory responses in macrophages and has been considered a crucial regulator of TLR receptors and NF-κB signaling in macrophages [72]. This prompted us to evaluate the role of TLR2 and TLR4 activation in NO production using macrophage cell lines derived from the bone marrow of TLR2-knockout mice, and macrophage cell lines derived from the bone marrow of TLR4-knockout mice. Interestingly, we observed that the production of NO by strains 219, 367, and 370 is strictly related to TLR2 and TLR4. Surprisingly, strain 366 triggered NO production in both TLR2 and TLR4–deficient BMDM cells, which indicates that there are some other receptors activated by this strain, providing NO production in macrophages, directly or indirectly. Other strains: 218, 368, 369, 371, 372, and 373 activated only TLR4-dependent signaling pathway (Fig. 3). The *Bifidobacterium* cell surface is abundant in plenty of antigens that include, i.e., polysaccharides, proteins, (lipo) teichoic acids, and glycolipids. Those molecules can activate TLR receptors and thus induce NO production. However, TLR receptors are not the only ones responsible for this effect. NOD2 surface receptors that are associated with peptidoglycan recognition can also contribute to NO production [73]. This might explain the NO production in TLR2 and TLR4 deficient cells after 366 stimulation. Moreover, LTA produced by gram-positive bacteria (including *Lacticaseibacillus* species or *Staphylococcus aureus*) induces NO production through different mechanisms including TLR2 receptor and Myd88-dependent signaling pathway [74].

It is proven that both probiotic and commensal bacteria do not trigger a pro-inflammatory response against each other; however, they may induce non-specific immune mechanisms responsible for maintaining immunological homeostasis and combating the pathogenic factors [75–78]. Additionally, probiotics regulate functions of the hosts’ immune cells including macrophages, facilitating their polarization towards the M1 phenotype. M1 macrophages can control infection by releasing a wide spectrum of factors including IL-1β, TNF-α, IL-6, and iNOS expression [79]. Induction of nitric oxide production in the gut epithelial cells and macrophages might be one of the beneficial functions of probiotic *Bifidobacterium* strains; thus, they protect the intestinal microenvironment and control the intestinal barrier permeability. As was presented in the literature, NO, depending on the source of origin, can play diversified roles. For instance, NO generated by cancer cells induces carcinogenesis, whereas those produced by myeloid cells impact the CD8 + T-cells and help to eliminate cancer [80]. Similarly, virally infected cells can induce the production of NO directly (by the virus replication in infected cells) and through an indirect path (infections mediators induce its production by the host cells and activate the innate immune response) [38, 39, 81]. Nitrates supplied with food and those produced within the digestive system are extremely toxic to intestinal pathogenic bacteria such as *Escherichia coli*, *Salmonella enterica*, and other pathogenic bacteria [32]. Nevertheless, the role of NO in the process of intestinal inflammation is still controversial. NO may restrain lymphocyte proliferation, leukocyte infiltration, and adhesion and may also protect against mucosal injury [82, 83]. On the other hand, excessive NO production can be associated with inflammation and apoptosis and may cause increased epithelial permeability [84]. There is a lot of evidence indicating that the physiological level of NO may protect gut mucosa by regulating mucosa blood flow or by inhibiting the primary steps of inflammation [85]. It was also observed in the iNOS-knock-out mice that NO produced by iNOS plays a critical role in the protective response to injury in intestinal inflammation and the healing process [85]. However, it is still very challenging to determine which concentration of released NO can be considered “physiological” and safe for the gut environment.

However, this study has some limitations that should be discussed. Firstly, it has not been investigated whether the level of NO released depends on *Bifidobacterium* CFU/mL, and only one dose, 0.5 × 10^8^ CFU/mL was tested. Secondly, we used the full bacteria to stimulate BMDM cells, but not particular mediators secreted by *Bifidobacterium* or their vesicles. Analysis of the biological activity of the *Bifidobacterium-*secreted mediators could be very desirable to check what is the main stimulator of TLR-dependent NO production. Also, there were no experiments performing preincubation of BMDM cells with MAP kinases inhibitors to show direct inhibition of iNOS expression by Western blotting. Determinations were limited mainly to checking the impact of U0126 and SP600125 inhibitors on nitric oxide production. Numerous lines of studies revealed that the NO and its downstream reactive nitrogen intermediates (RNIs) are toxic to microbes and host cells via cysteine S-nitrosylation of proteins, deamination of nucleic acids, or desaturation of lipids [86]. So, it would be interesting to check if the NO amounts released by BMDM cells in response to *Bifidobacterium* strains exert a toxic effect on some pathogenic bacteria.

In conclusion, probiotic *Bifidobacterium* strains of human origin are successfully used both in the prevention and treatment of colitis as well as other gastrointestinal disorders [87]. The fact that *Bifidobacterium* possesses the ability to modulation of macrophage activity (induction of NO production in “healthy” macrophages and inhibition of NO production in LPS-pretreated macrophages) indicates their immunological and cytoprotective functions. The present study provides evidence that the immunoprotective activity of *Bifidobacterium* on the host cells could lay in macrophages’ activation. It is pronounced by the production of iNOS-dependent NO by activating MAPK/NF-κB signaling pathway. These effects could be mediated by the bacterial cell wall or cytoplasmic components affecting macrophages via receptors present on their surface. These findings might give a new perspective into the function of *Bifidobacterium* in regulating the host immune defense against pathogen invasion. However, further studies are needed to investigate in detail the effect of *Bifidobacterium-*mediated macrophage activation and polarization against pathogen infection.

## Data Availability

The datasets used and/or analyzed during the current study are available from the corresponding author upon reasonable request.
